# Peri-Transplant Psychosocial Factors and Neutrophil Recovery following Hematopoietic Stem Cell Transplantation

**DOI:** 10.1371/journal.pone.0099778

**Published:** 2014-06-10

**Authors:** Jennifer M. Knight, Jan A. Moynihan, Jeffrey M. Lyness, Yinglin Xia, Xin Tu, Susan Messing, Bryan C. Hunter, Li-Shan Huang, Rosemary O. Obi, D'Arcy Gaisser, Jane L. Liesveld, Olle Jane Z. Sahler

**Affiliations:** 1 University of Rochester Medical Center, Department of Psychiatry, the Rochester Center for Mind-Body Research, Rochester, New York, United States of America; 2 University of Rochester Medical Center, Department of Biostatistics and Computational Biology, Rochester, New York, United States of America; 3 Nazareth College, Department of Music Therapy, Rochester, New York, United States of America; 4 University of Rochester Medical Center, Department of Pediatrics, Division of Hematology/Oncology, Rochester, New York, United States of America; 5 University of Rochester Medical Center, Department of Cardiology, Rochester, New York, United States of America; 6 University of Rochester Medical Center, Department of Medicine, Division of Hematology/Oncology, Rochester, New York, United States of America; 7 University of Rochester Medical Center, Department of Psychiatry, Rochester, New York, United States of America; 8 University of Rochester Medical Center, Department of Medical Humanities, Rochester, New York, United States of America; Wake Forest Institute for Regenerative Medicine, United States of America

## Abstract

**Objective:**

Multiple psychosocial factors appear to affect cancer progression in various populations; however, research investigating the relationship between psychosocial factors and outcomes following hematopoietic stem cell transplantation (HCT) is scarce. Subject to adverse immunological and psychological conditions, HCT patients may be especially vulnerable to psychosomatic health sequelae; therefore, we studied whether optimism and anxiety influence the pertinent clinical outcome of days to neutrophil engraftment (DTE).

**Method:**

54 adults undergoing either autologous or allogeneic HCT completed self-report questionnaires measuring optimism and anxiety. We assessed the association between these psychosocial variables and DTE.

**Results:**

Greater optimism and less anxiety were associated with the favorable outcome of fewer DTE in autologous HCT recipients, though this relationship was no longer significant when reducing the sample size to only subjects who filled out their baseline survey by the time of engraftment.

**Conclusion:**

Our findings are suggestive that optimism and anxiety may be associated with time to neutrophil recovery in autologous, but not allogeneic, adult HCT recipients. Further investigation in larger, more homogeneous subjects with consistent baseline sampling is warranted.

## Introduction

Psychosocial factors affect cancer progression in multiple populations [1]–[3]. However, research investigating these relationships following hematopoietic stem cell transplantation (HCT) is scarce, with few studies investigating the differential effect of emotions on outcomes and immune recovery [4]–[9]. The number of transplants continues to increase, with 8,000 allogeneic and 11,000 autologous HCTs performed in the United States in 2011 [10]. Their use, safety, efficacy, and availability for hematologic malignancies and disorders have greatly expanded, making it important to identify positive and negative prognostic factors and immunologic mechanisms in this population.

HCTs are associated with unique immunological and psychological conditions. Patients receiving transplants are more immunosuppressed than other cancer patients secondary to ablation of their native marrow. Furthermore, the clinical course of HCT may be more distressing than other cancer treatments, with potentially prolonged periods of physical and emotional isolation depending on the type of transplant, institutional practice, and potential complications [9]. There is generally a significant decline in quality of life in the immediate post-transplant period [11].

Positive psychosocial variables such as optimism predict not only emotional, but also physical well-being after HCT, including survival [6], [12]. The psychoneuroimmune mechanisms mediating these associations are unknown. Negative pre-transplant emotions, including depression and anxiety, have been associated with worse post-transplant outcomes and survival [4], [5], [7], [13]; however, other studies have not confirmed this association [14], [15].

Neutropenia is a major contributor to morbidity following transplant [16]; the longer the period of neutropenia, the greater the potential for infectious complications [17]. Conversely, survival is improved with earlier neutrophil engraftment [18]. Days to neutrophil engraftment (DTE) may, therefore, contribute to differences in mortality among HCT patients with differing pre-transplant psychological profiles. Recently, anxiety and depression were observed to be associated with slower immune recovery as measured by white blood cell (WBC) count following autologous transplant [7]. This relationship has never been examined in allogeneic recipients.

We investigated the association of optimism and anxiety with time to neutrophil recovery in a population of adult HCT recipients undergoing either autologous or allogeneic transplant. We hypothesized that greater optimism and less anxiety around the time of transplant would be associated with less time to neutrophil recovery.

## Methods

### Participants

Patients entered the study between Day −20 and Day +3, with Day 0 being the day of stem cell transplant. The goal was to enroll participants as close to Day −8 as possible, but preferably at least prior to transplant. Most commonly, enrollment occurred at the last pre-transplant outpatient visit prior to transplant. Reasons for variation in enrollment time included lack of access to the patient at their pre-transplant visit due to patient, provider, or study staff conflicts or overlap. If a patient was unable to be approached at a pre-transplant visit, they were approached as soon as possible following hospital admission for transplant. On occasion this resulted in enrolling participants after their transplant if admission occurred on a Friday and the conditioning period was short, as occurs with multiple myeloma (conditioning with high dose melphalan on Day −1). One hundred and twenty-two patients were approached by a member of the research team for study participation; 64 (52%) agreed to participate and 54 provided complete psychosocial data. Any patient admitted to the Bone Marrow Transplant Program at the University of Rochester Medical Center for transplantation of any type, age ≥18 years of age or older and able to give informed consent and complete the study questionnaires was eligible to participate. There were no medical condition exclusions. Factors noted to affect neutrophil recovery rates including age, race, gender, conditioning regimen (myeloablative/non-myeloablative), stem cell source (bone marrow or peripheral blood), and number of CD34^+^ cells infused/kg of recipient body weight were controlled for. Allogeneic and autologous transplants were evaluated separately. All participants received homogeneous granulocyte colony stimulating factor (GCSF); therefore, it was not necessary to include GCSF as a covariate. Due to the small number of minorities, race was dichotomized as Caucasian or non-Caucasian.

### Psychological Measures

Upon obtaining written informed consent (as close to Day −8 as possible), a member of the research team provided participants with the self-administered surveys and was available as long as necessary to answer any questions and ensure participants understood how to complete them. Participants were asked to complete the surveys independently to avoid any possible influence from the research staff. They were also asked to complete them as soon as possible to be picked up the following day by a research team member. There was variation in the time of survey completion, ranging from Day −20 to Day +32. Fifty-seven percent of subjects completed the surveys prior to transplant, 82% by Day +3, and 85% by Day +14.These variations were due to either or both a) variation in time of study enrollment or b) a delay in participants filling out the survey on their own and returning it. Reasons for delays in enrollment are noted as above. The psychological parameters assessed were:

#### Optimism

The Life Orientation Test (LOT) assesses individual differences in generalized optimism versus pessimism. The 10-item revised version (LOT-R) [19] focuses explicitly on expectations for the future (scored 0–24).

#### Anxiety

The 20-item State-Trait Anxiety Inventory, Trait Version (STAI-T) [20] was assessed in this study (scored 20–80). Only the trait component of the STAI was evaluated as we were most interested in the effects of anxiety as a stable personality trait. This measure was also chosen due to the more consistent relationship between trait anxiety and changes in immune function [21]–[24] rather than the more ambiguous relationship between state anxiety and immune function [25]–[27].

These psychosocial measures were utilized as part of a music therapy intervention study in which 32 participants were randomized to the music therapy intervention and 32 randomized to an expressive reading arm; both groups received the same time and attention support. Other psychosocial measures evaluated as part of the initial study included coping style, social support, locus of control, and religiousness. We chose to evaluate optimism and anxiety due to their availability and evidence in the literature to support their relationship with transplant outcomes.

### Transplant Engraftment

DTE was measured as the day post-transplant that the absolute neutrophil count (ANC) was >500/mm^3^ for ≥3 consecutive days, a clinically meaningful endpoint at which time the risk for serious bacterial or fungal infections starts to decrease [28].

### Statistical Analysis

Data are expressed as means ± SD for continuous variables and as the number (percentage) for categorical variables. DTE also included the range for each transplant type. Comparisons of patient characteristics for the transplant types were 2-sided and included the use of t-tests, chi-square, or Fisher's exact test, as appropriate to the data. P values of <.05 were considered to be statistically significant. Multiple regression analyses were conducted using a general linear model program. The analyses were carried out separately for autologous and allogeneic transplants. The outcome DTE was analyzed by optimism and anxiety separately while controlling both demographic and clinical covariates of age, race, gender, conditioning regimen, stem cell source, and number of CD34^+^ cells. T-tests assessed the differences for autologous and allogeneic transplants in optimism and anxiety overall as well as for those who filled out their survey by Day +3 and those who did not. Analyses were additionally run for only those participants who filled out their survey by Day +3, effectively reducing any bias that knowledge of engraftment status might have on perceived anxiety or optimism. All analyses were carried out using SAS/STAT software, Version 9.3 of the SAS System (Copyright © July, 2011, SAS Institute Inc) on a Windows 7 platform.

## Results

Patients' pre-transplant diagnoses included lymphomas (N = 27; 42.19%), leukemias (N = 21; 32.81%), solid tumors (N = 2; 3.13%), multiple myeloma (N = 10; 15.63%), and other hematologic disorders (4; 6.25%). Table 1 presents the baseline descriptive characteristics for the 64 patients (33 men, 31 women) participating in the study. Reported optimism levels had a mean and SD of 16.44 (5.38) and range of 4–24 while reported anxiety levels were 38.44 (9.22) with a range of 24–59. There was no significant difference in levels of anxiety or optimism between autologous and allogeneic recipients (p = 0.89 and p = 0.71, respectively). These traits were very highly negatively correlated (Pearson r = −0.70, p<0.0001). While the initial study was a randomized controlled trial evaluating the efficacy of music therapy, both treatment arms were an active intervention (music therapy vs. expressive reading), and there were no differences in psychosocial or other outcomes between groups.

**Table 1:** Participant characteristics, N = 64.

	Autologous (N = 33)	Allogeneic (N = 31)	p-value*
Age at transplant, mean (SD)	51.03 (10.53)	42.77 (13.26)	<0.008
Race, N (%)			0.078
Caucasian	31 (93.94)	24 (77.42)	
Non-Caucasian	2 (6.06)	7 (22.58)	
Gender, N (%)			0.321
Female	14 (42.42)	17 (54.84)	
Male	19 (57.58)	14 (45.16)	
Conditioning regimen, N (%)			<0.0001
Myeloablative	32 (96.97)	15 (48.39)	
Non-Myeloablative**	1 (3.03)	16 (51.61)	
Stem cell source, N (%)			0.002
Bone Marrow	0 (0)	8 (25.81)	
* Sibling*		*2 (25)*	
* MUD* ***		*6 (75)*	
Peripheral Blood	33(100)	23 (74.19)	
* Sibling*		*20 (86.96)*	
* MUD* ***		*3 (13.0)*	
Diagnosis			<0.0001
Lymphomas	22 (66.67)	5 (16.13)	
Leukemias	0 (0)	21 (67.74)	
Other hematologic disorders	0 (0)	4 (12.90)	
Solid tumors	1 (3.03)	1 (3.23)	
Multiple myeloma	10 (30.30)	0 (0)	
Number of CD34^+^ cells infused/kg of recipient body weight, mean (SD)	4.40 (1.68)	3.73 (1.80)	0.127
Days to engraftment, mean (SD; range)	13.48 (2.24; 6–18)	18.00 (4.76; 11–30)	<0.0001

SD = Standard deviation.

*Determined by t-test, chi-square, or Fisher's exact test, as appropriate to the data.

**Non-myeloablative patient in autologous group may be a reporting error but unable to verify.

***MUD = matched unrelated donor.

Greater optimism and less anxiety were significantly associated with fewer DTE among autologous HCT recipients; however, these psychosocial factors were not significantly associated with DTE when evaluating the more restricted sample of participants who filled out their surveys by Day +3 (Table 2). There was no significant association between optimism or anxiety and DTE among allogeneic recipients collectively (β = −0.0059, p = 0.97; β = −0.1446, p = 0.11, respectively) or for only those who filled out their surveys by Day +3 (β = −0.0994, p = 0.68; β = −0.1866, p = 0.27, respectively). There were no significant differences for either autologous or allogeneic transplant recipients in scores on the anxiety or optimism measures when compared by time of survey completion (Table 3).

**Table 2:** Multivariate results of optimism or anxiety and covariates associated with days to neutrophil engraftment in autologous hematopoietic stem cell transplant recipients.

	All Participants (N = 31)			Participants who filled out survey by Day +3 (Optimism N = 23; Anxiety N = 26)		
	Estimate (β)	SE	*P* - value	Estimate (β)	SE	*P* - value
**Optimism**	−0.15	0.06	**0.01**	−0.09	0.11	0.44
Age	0.02	0.03	0.59	0.04	0.06	0.57
Race (Caucasian)*	−0.33	1.53	0.83	-**	-**	-**
Gender (Female)*	1.12	0.71	0.12	0.99	1.26	0.45
Conditioning regimen	−2.19	1.60	0.18	−2.32	2.88	0.43
CD34^+^ cells infused	−0.14	0.19	0.48	−0.12	0.35	0.74
**Anxiety**	0.08	0.04	**0.02**	0.07	0.06	0.27
Age	0.04	0.03	0.26	0.04	0.06	0.46
Race (Caucasian)*	−0.58	1.64	0.73	−0.82	2.80	0.77
Gender (Female)*	0.91	0.72	0.21	0.41	1.14	0.72
Conditioning regimen	−2.87	1.56	0.07	−2.28	2.62	0.40
CD34^+^ cells infused	−0.09	0.19	0.64	−0.12	0.32	0.71

*Category coded 1 (vs. 0) indicated in parentheses.

**All participants were white.

**Table 3:** Anxiety and optimism scores by type of transplant and as a function of time of survey completion.

	All Participants		Autologous		Allogeneic*	
	Autologous (N = 33)	Allogeneic (N = 31)	Participants who filled out survey ≤ Day +3	Participants who filled out survey > Day +3	Participants who filled out survey ≤ Day +3	Participants who filled out survey > Day +3
Anxiety Score (SD)	38.60 (8.51)	38.25 (10.24)	38.85 (8.47)	37.00 (9.90)	38.81 (10.17)	38.50 (10.47)
	N = 30	N = 24	N = 26	N = 4	N = 16	N = 4
			p = 0.69		p = 0.96	
Optimism Score (SD)	16.18 (5.53)	16.73 (5.30)	15.65 (5.40)	18.60 (6.11)	16.06 (5.25)	16.20 (6.02)
	N = 28	N = 26	N = 23	N = 5	N = 17	N = 5
			p = 0.29		p = 0.96	

*Differences in N reflect missing date of survey completion.

## Discussion

We partially confirmed our hypothesis: greater optimism and less anxiety in the peri-transplant period were significantly associated with fewer DTE in autologous HCT recipients, although this relationship was no longer significant with the reduced sample size evaluating only subjects who completed their survey by Day +3. The effect remained similar, however, as indicated by the standardized estimates. Fewer DTE would be expected to result in a shorter period of extreme infectious susceptibility and increased survival.

These results suggest one possible mechanistic explanation to the growing body of literature on the effects of psychosocial factors on HCT outcomes [4], [8], [9], [29]; however, due to the nature of this study and our limited sample size, this is purely associational data that should prompt further exploration in larger, more homogeneous samples. Longer DTE may be an early event in the dysregulated immunological cascade predisposing psychosocially-impaired transplant recipients to higher levels of morbidity and mortality. The exact mechanism linking psychological factors and neutropenia remains to be elucidated and is likely not straightforward. Acute stress and inflammatory cytokines can actually induce early mobilization of neutrophils [30], [31], though this effect may be enhanced or impaired pending exposure to different sex hormones [32]. Also in support of the adverse effects of psychological stress, catecholamines decrease function and phagocytic capabilities of neutrophils [33], [34]; therefore, it may be necessary to measure function and not simply absolute neutrophil numbers in future studies. Finally, differences in effect on neutrophils between acute and chronic psychosocial states will need to be evaluated when elucidating this mechanism.

We did not observe significant findings with allogeneic transplant patients; we suggest that the additional significant physiological burdens involved in their treatment might diminish the impact of psychosocial factors on DTE for this group. Another possibility is that the psychosocial effect may be somehow imprinted on the transplanted cells themselves, preventing the observation of this effect when the donor psychosocial status is unknown, as occurs in this study with allogeneic transplantation. Finally, allogeneic recipients take immunosuppressive agents to mitigate graft versus host disease; these may also mask the relationship between psychosocial factors and immune outcomes.

Our results are consistent with prior work demonstrating an impact of anxiety on immune recovery post-transplant [7]. We extended this previous work by investigating the relationship among allogeneic recipients and also examining optimism as an additional psychosocial factor of importance. Our assessment of immune recovery was slightly different; MgGregor et al [7] evaluated WBC counts over days 5–22, while we looked at the more specific WBC subset of interest during immediate immune recovery – neutrophils – and evaluated an endpoint used in clinical practice - DTE. In the only previous study evaluating optimism and transplant outcome, pre-transplant optimism predicted better survival in the first two months post-HCT, but not at six months [6]. Our results provide one explanation for the dissipation of this effect on survival over time, as neutrophil engraftment is a more proximal event.

There are some significant limitations to our study. We investigated a very heterogeneous group (i.e., diagnoses, baseline levels of illness and supportive care); future analyses should either be powered to control for these differences or examine a more homogeneous group. Our most significant limitation is the variability in timing of when the baseline surveys were completed, which when restricted to those who filled out their surveys by the time of engraftment resulted in psychosocial factors and DTE not being significantly associated. This could mean that in fact knowledge of engraftment influenced optimism and anxiety; however, there are three reasons why this knowledge may not have significantly influenced anxiety or optimism in our study. First, we evaluated trait and not state anxiety, with studies demonstrating substantial stability of both the short- (20–100 days) and long-term (years) STAI-T measure [35]. The LOT-R also tends to evaluate dispositional or “trait” optimism [36]. Traits are less subject to influence by a discrete event. Second, none of the anxiety or optimism scores between or among autologous and allogeneic recipients were significantly different as a function of time of survey completion, albeit we did have a small sample within which to detect a difference. Third, there are a multitude of other physical and medical factors and complications that could also have arisen during that time period to reasonably affect psychosocial status. Alternatively, it is possible that with a restricted sample size we simply lost power to detect significance; this may be the case given that our results were similar in direction and size while no longer significant. Our findings therefore should be considered preliminary and should be replicated in a larger sample with more consistent timing of the baseline survey before more definitive conclusions may be drawn.

In conclusion, our findings suggest that optimism and anxiety may be associated with time to neutrophil recovery in autologous stem cell transplant patients, though this relationship was no longer significant with the reduced sample size evaluating subjects completing their psychosocial surveys by Day +3, with more favorable peri-transplant psychosocial factors associated with shorter time to neutrophil recovery. Further research is needed with larger and more homogeneous cohorts of HCT patients with consistent baseline sampling to investigate the impact of psychosocial factors on DTE and other immunologic transplant sequelae with the goal of improving outcomes in this physically and psychologically stressed population.
